# Rings and Bricks: Expression of Cohesin Components is Dynamic during Development and Adult Life

**DOI:** 10.3390/ijms19020438

**Published:** 2018-02-01

**Authors:** Laura Rachele Bettini, Federica Graziola, Grazia Fazio, Paolo Grazioli, Valeria Scagliotti, Mariavittoria Pasquini, Giovanni Cazzaniga, Andrea Biondi, Lidia Larizza, Angelo Selicorni, Carles Gaston-Massuet, Valentina Massa

**Affiliations:** 1 Dipartimento di Scienze Della Salute, San Paolo Hospital Medical School, Università degli Studi di Milano, 20142 Milan, Italy; laurarbettini@gmail.com (L.R.B.); federica.graziola@fastwebnet.it (F.G.); paolo.grazioli@unimi.it (P.G.); marvi.pasquini@gmail.com (M.P.); 2 Clinica Pediatrica, Dipartimento di Medicina e Chirurgia, Università di Milano-Bicocca Ospedale San Gerardo/Fondazione MBBM, 20900 Monza, Italy; andrea.biondi@unimib.it; 3 Centre for Endocrinology, William Harvey Research Institute, Barts and the London School of Medicine and Dentistry, Queen Mary University of London, London EC1M 6BQ, UK; v.scagliotti@qmul.ac.uk (V.S.); c.gaston-massuet@qmul.ac.uk (C.G.-M.); 4 Centro Ricerca M. Tettamanti, Clinica Pediatrica, Dipartimento di Medicina e Chirurgia, Università di Milano-Bicocca, Ospedale San Gerardo/Fondazione MBBM, 20900 Monza, Italy; grazia.fazio@unimib.it (G.F.); gianni.cazzaniga@asst-monza.it (G.C.); 5 Laboratory of Medical Cytogenetics and Molecular Genetics, IRCCS Istituto Auxologico Italiano, 20154 Milan, Italy; lidia.larizza@unimi.it; 6 UOC Pediatria, ASST Lariana, 22077 Como, Italy; angelo.selicorni61@gmail.com

**Keywords:** cohesin complex, central nervous system, hematopoietic tissues, gene expression

## Abstract

Cohesin complex components exert fundamental roles in animal cells, both canonical in cell cycle and non-canonical in gene expression regulation. Germline mutations in genes coding for cohesins result in developmental disorders named cohesinopaties, of which Cornelia de Lange syndrome (CdLS) is the best-known entity. However, a basic description of mammalian expression pattern of cohesins in a physiologic condition is still needed. Hence, we report a detailed analysis of expression in murine and human tissues of cohesin genes defective in CdLS. Using both quantitative and qualitative methods in fetal and adult tissues, cohesin genes were found to be ubiquitously and differentially expressed in human tissues. In particular, abundant expression was observed in hematopoietic and central nervous system organs. Findings of the present study indicate tissues which should be particularly sensitive to mutations, germline and/or somatic, in cohesin genes. Hence, this expression analysis in physiological conditions may represent a first core reference for cohesinopathies.

## 1. Introduction

The structure of the cohesin complex has been well described in all metazoa, showing a high degree of conservation in the number of components, protein structure and assembly dynamics [1,2,3].

On the other hand, complex functions have been increasingly unraveled. The canonical function was first described in the budding yeast, *Saccharomyces cerevisiae* [4,5,6,7] and was later confirmed in all studied organisms, including mammals [8]. This function is essential for cell division, as cohesins hold sister chromatids together until anaphase, when the mitotic spindle separates DNA content in daughter cells.

Subsequently, two additional functions, termed “non-canonical” have been put forward. The first highlighted an essential role for the cohesin complex in DNA repair, both by homologous recombination and check-point induction [9,10]. The second, by which the cohesin ring intervenes in gene expression regulation both by three-dimensional chromatin folding and RNA polymerase recruitment, grounded on in vivo genetic data on *Drosophila melanogaster* and genome-wide studies in fly cultured cells [11] and was then confirmed in vertebrate models [12].

Mutations in members of the cohesin complex have been associated with genetic developmental disorders with intellectual disability named cohesinopathies, of which Cornelia de Lange syndrome (CdLS) is the most frequent and best known entity [13,14]. CdLS is a malformative syndrome affecting many organs, including central nervous system, gastrointestinal and musculoskeletal [15] CdLS is genetically heterogeneous (CdLS1 MIM 122470, CdLS2 MIM 300590, CdLS3 MIM 610759, CdLS4 MIM 614701, CdLS5 MIM 300882), with a broad clinical expressivity and up to 80% of cases carry heterozygous autosomal or X-linked mutations in one of cohesin complex components/regulators: *SMC1A*, *SMC3*, *RAD21*, *NIPBL*, *HDAC8* [16].

In the last decade, various organisms (fruit fly, zebrafish and mouse) have been used for modeling cohesionopathies and functional studies have highlighted the extensive transcriptional dysregulation caused by different CdLS pathogenic variants [17,18,19]. Surprisingly, a basic description of the mammalian expression pattern of the main CdLS causative genes in physiologic condition is still missing. Hence, for better forwarding the challenge of clinical observations and interpretation of the models-phenotype correlations, here we report a detailed analysis of expression in murine and human tissues of the cohesin genes which are defective in CdLS.

## 2. Results

### 2.1. Cohesin Genes Are Ubiquitously and Differentially Expressed in Human Tissues

*NIPBL*, *SMC1A*, *SMC3*, *RAD21* and *HDAC8* expression was detected in all analyzed fetal (liver and brain) and adult tissues (heart, lung, kidney, adrenal gland, salivary gland, trachea, small intestine, stomach, thyroid, spleen, thymus, brain, cerebellum, bone marrow and peripheral blood). Expression analysis was normalized using two different tissues (heart and adrenal gland) as calibrator and the same results were obtained. In particular, the expression profile varied across the five genes and for each of them across different organs. Figures S1 and S2, and Table S1 provide the results normalized on heart. Kidney and respiratory tract showed scarce expression for all cohesin subunits/regulator genes whereas the gastrointestinal tract showed robust gene expression levels. All genes share high or peak expression levels in hematopoietic and cerebellum (see next sections). The global expression profiling of the five genes was comparable in all tissues, with the exception of *NIPBL* which showed the highest expression levels. Comparing our results with data publicly available (https://www.ebi.ac.uk/gxa/home), although expressed in different units and obtained with other techniques and less detailed, the trend of expression was comparable (Tables S2–S6). Interestingly, organs frequently affected in CdLS patients showed abundant expression of causative cohesin genes (Table S7).

### 2.2. Cohesins Expression Is Intense in Hematopoietic Tissues 

**Figure 1:**
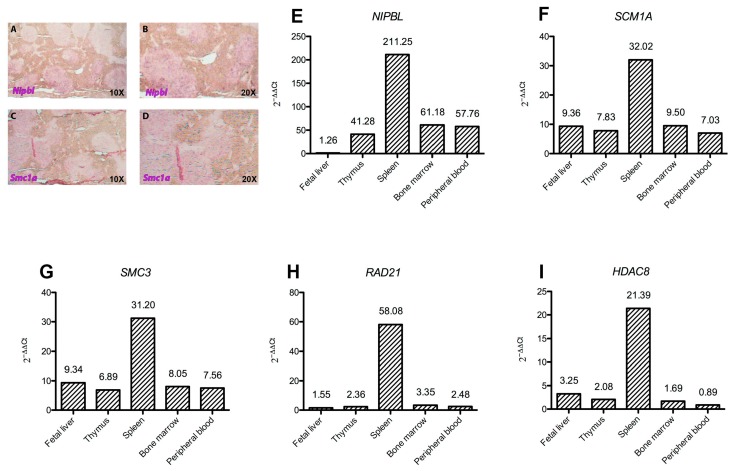
Cohesin expression in hematopoietic tissues. Histograms (**E**–**I**) show *NIPBL*, *SMC1A*, *SMC3*, *HDAC8* and *RAD21* gene expression levels in fetal liver, thymus, spleen, bone marrow and peripheral blood as 2^−ΔΔ*C*t^. In situ hybridization on 10 micron-sections of (**A**,**B**) lower and higher magnification and (**C**,**D**). Both genes are expressed in spleen follicles of wild-type animals as positively stained cells. *Nipbl* expression appears scattered in the follicles, rich in lymphocytes (**A**,**B**), whereas *Smc1a* (**C**,**D**) localizes particularly in the germinative area of the follicle.

All human hematopoietic tissues analyzed—fetal liver, thymus, spleen, bone marrow, and peripheral blood- displayed high levels of *NIPBL*, *SMC1A*, *SMC3*, *RAD21* and *HDAC8* expression. In particular, the spleen showed the most abundant levels, ranging from 20 folds (*HDAC8*) to 200 folds—*NIPBL*—increased expression. On the other hand, an opposite expression pattern was observed in the fetal liver, whereas *HDAC8* and *RAD21* showed the lowest levels of expression in the peripheral blood (Figure 1). 

Using spleens from adult wild-type mice, *Nipbl* and *Smc1a* distribution was assessed by in situ hybridization on paraffin sections. The expression of both genes could be visualized in spleen follicles of wild-type animals as positively stained cells. *Nipbl* expression appears scattered in the follicles (Figure 1A,B), rich in lymphocytes, whereas *Smc1a* seems to localize especially in the germinative area of the follicle (Figure 1C,D).

### 2.3. Cohesins Are Differentially Expressed in Bone Marrow Cells

**Figure 2:**
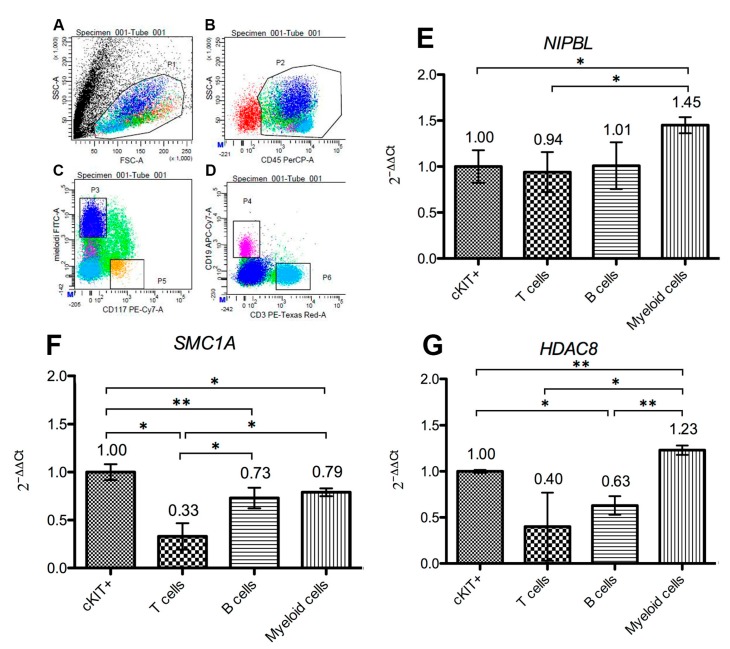
Analysis of bone marrow cell subpopulations. (**A**–**D**) FACS cell sorting analysis of subpopulations was achieved with specific antibodies recognizing c-KIT/CD117 (correspondent to multipotent progenitor cells), myeloid cell antigens (antibodies mix anti-CD15 and anti-CD33), CD3 (to select T lymphocytes) and CD19 to detect B lymphocytes, respectively. As a hierarchical gating strategy, the four cell subsets were selected on CD45 positive population, which represents a pan-leukocyte antigen, to identify white blood cells. Dot plots of dimensional scatters (FSC and SSC) are shown, in addition to sorting gates, P3 (myeloid cells), P4 (B cells), P5 (precursors) and P6 (T cells). Histograms (**E**–**G**) show *SMC1A* core subunit, *NIPBL* loading factor and *HDAC8* regulator gene expression as 2^−ΔΔ*C*t^ in stem cells (cKIT+), T cells, B cells and myeloid cells. Error bars represent standard deviation. Asterisks indicate statistically significant values: * *p* ≤ 0.05; ** *p* ≤ 0.005.

Gene expression analysis in FACS-sorted bone marrow cell subpopulations revealed a dynamic cell-specific expression. In particular, the genes for cohesin complex *SMC1A* core subunit, *NIPBL* loading factor and *HDAC8* regulator, are expressed in all cell types, including KIT-positive progenitor cells, B cells, T cells and myeloid population, with differences among genes and among cells (Figure 2). Nevertheless, for all analyzed genes, the myeloid compartment exhibited the most abundant expression, where the relatively most abundant gene is *SMC1A*, compared to both *HDAC8* and *NIPBL*.

### 2.4. Cohesins Are Differentially Expressed in Central Nervous System Tissues

**Figure 3:**
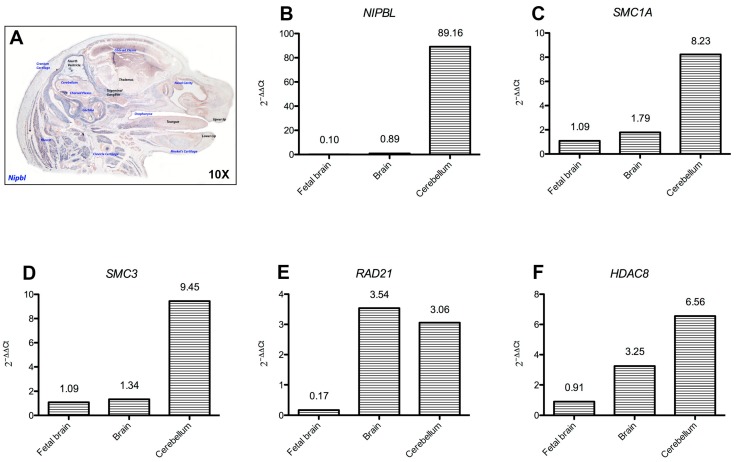
Cohesin expression in the central nervous system. (**A**) In situ hybridization on 10 micron-sections of 17.5 dpc (days post coitum) mouse fetus, revealed *Nipbl* expression as dynamic in the fetal CNS. Structures found positive for *Nipbl* expression are shaded in blue. (**B**–**F**) Histograms show *NIPBL*, *SMC1A*, *SMC3*, *HDAC8* and *RAD21* gene expression levels in human fetal brain and human adult brain and cerebellum as 2^−ΔΔ*C*t^.

**Figure 4:**
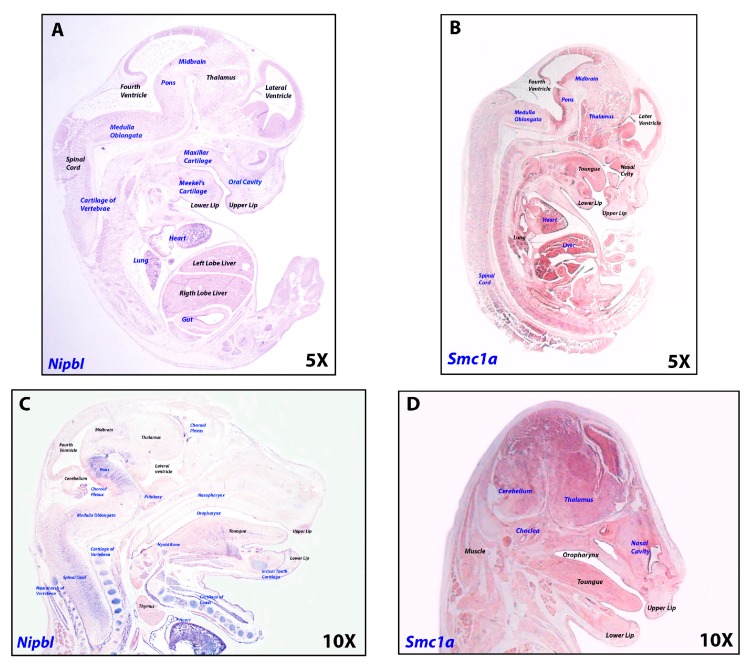
*Nipbl* and *Smc1a* expression is modulated during development. In situ hybridization on 10 micron-sections of 13.5 dpc (days post coitum) (**A**,**B**), 15.5 dpc (**C**,**D**) mouse fetuses, revealed *Nipbl* and *Smc1a* expression as dynamic during development. Structures where *Nipbl* and *Smc1a* were found to be expressed are in blue.

All cohesins were found to be expressed in brain, cerebellum and fetal brain, with *NIPBL, SMC1A*, *SMC3* and *HDAC8* highly expressed in the cerebellum (Figure 3). Analysis of mouse developing brain by in situ hybridization of *Nipbl* revealed a dynamic pattern of expression at different developmental stages (Figure 3 and Figure 4 and Table S8). *Smc1a* expression was similar to *Nipbl* in showing gene expression distribution augmenting with developmental age in all fetal tissues, especially at the level of hindbrain-derived neural structures, such as pons, medulla oblongata, choroid plexuses and cerebellum.

## 3. Discussion

CdLS is a rare mendelian developmental intellectual disability disorder characterized by genetic heterogeneity and variable severity of adverse phenotype, caused by mutations in genes coding for the cohesin complex. Notwithstanding the multiple functions of the cohesin complex in the fundamental processes of chromosome segregation and post-replicative DNA repair [5,9], a large body of evidence has fostered the current assumption that defect in the “non-canonical function” of regulation of gene expression is the main driving pathomechanism of cohesinopathies [10,20,21,22,23]. Several pathways with a known fundamental role in development have been found to be disrupted in cohesinopathies models [19,21,24,25,26]. However, a clear description of quantitative expression and pattern of the cohesin genes in adult and fetal tissues from control subjects is a relevant premise to seek for associations of altered pathways with clinical features underpinning the variable phenotype within and across CdLS subentities. Prompted by the scarce data in the literature, we sought to analyze the transcripts of the cohesin complex genes which mutations underlie CdLS subtypes, to assess their levels and distribution across different tissues. Quantitative RT-PCR analysis evidenced a ubiquitous, although differential expression of the cohesin genes. In particular, we found cohesins expressed not only in tissues with intense proliferative activity and renewal turnover (such as the bone marrow and the peripheral blood), in keeping with the canonical role of this complex, but also in tissues with modest or absent cell proliferation such as the nervous system, as previously reported also in *D. melanogaster* [27].

As regards the sustained expression of all cohesin genes in the bone marrow, which has been deepened by assays on specific FACS-sorted bone marrow cell populations, we consider it may also reflect the cohesin role in controlling pluripotency factors. Indeed, the cohesin complex has been shown to regulate the adult hematopietic “stem cells pool” and it has been proposed as a master regulator of transcriptional cascade in hematopoiesis [28]. Accordingly, cohesin loss leads to myeloproliferative neoplasms in mice, outlining a tumor suppressing function for the cohesin complex in vivo [29]. The cohesin complex with the zing finger DNA-binding protein CTCF and Mediator have been identified as key players of chromatin loop organization [25,30]. Interestingly a contribution of cohesins, and Mediator to DNA looping specific of murine embryonic stem cells has been demonstrated by their interactions and co-occupancy of enhancer and core promoter regions of a set of genes active in ES (embryonic stem) cells, such as the pluripotency *Oct4*, *Sox2*, *Nanog* genes [25]. Cohesin genes are highly expressed in the myeloid compartment and this data is consistent with increasing evidences of cohesin somatic mutations in myeloid leukemia [31,32]. 

Moreover, in vivo modeling using *D. rerio* has demonstrated how *rad21* controls *runx1*, a master controller of hematopoiesis during embryogenesis [33,34]. RUNX1 has been shown to play a pivotal role in maintenance of homeostasis of hematopoietic stem and progenitor cells in mammals [35]. Aberrant expression of *RUNX1* is known to cause myeloid leukemia and since the first report in 2010 [36] a number of studies have shown somatic mutations in the cohesin complex as responsible for myeloid leukemia [32]. Consistently, differential expression of *Nipbl* and *Smc1a* was found in mouse spleen.

Moving to another likely relevant tissue/compartment, we showed that both fetal and adult cerebellum samples consistently express high levels of cohesin complex genes. As during adult life, the proliferative cells in this organ are limited, the non-canonical role of cohesin is postulated to be prominent in this district. Only a few indications of cerebellum-specific clinical findings emerge from the literature on cohesinopathies models and human patients [37]. Out of these, in *D. rerio*, both *smc1a* and *nipblb* haploinsufficiency result in abnormalities of the hindbrain, the embryonic precursor of the cerebellum [24,26] and it is interesting that out of the structures found to express cohesins during development, the majority were hindbrain-derived (as pons, medulla oblongata and choroid plexus).

CdLS patients do not normally present ataxia, however few reports on abnormalities of hindbrain derived structures have been published [38]. On the other hand, cerebellar hypoplasia is often found in syndromic patients, but the pathogenetic impact is still debated. Many studies have reported pathological findings in the cerebellum of patients diagnosed with autism and autism spectrum disorders, and cerebellum hypoplasia has also been correlated with non-syndromic delayed psychomotor development. It is worth noting that autistic features, such as repetitive behavior and expressive communication deficits, are often present in a subset of CdLS patients [39], bridging a monogenic and a multifactorial neurodevelopmental disorder. The clinical correlation is not yet validated, hence further studies should monitor anatomical abnormalities in the brain of CdLS patients aiming at better evaluating the clinical relevance of this data.

## 4. Materials and Methods 

### 4.1. Human Tissues

For a detailed analysis of gene expression, RNA from a commercially available biobank was used (Human Total Master Plan II, Clontech, Mountain View, CA, USA). Tissues were derived from healthy subjects, both males and females. In addition, total bone marrow (BM) cells from a single subject were collected, after permission by signed inform consent of healthy donor to donate leftover material from clinical procedures for research purposes. Ethics approval number 1739, approved by San Gerardo Hospital ethics committee on the 27 June 2013. Viable mononuclear cells were collected from the bone marrow of a healthy donors and purified by Ficoll-Paque density gradient (GE Healthcare Life Sciences, Marlborough, MA, USA), according to manufacturer’s protocol.

### 4.2. Antibodies and Flow Cytometry 

Peridinin chlorphyll protein (PerCP) anti CD45 (pan-leukocyte antigen), phycoerythrin-conjugated antibodies anti-CD117 (for precursors), fluorescein isothiocyanate- conjugated (FITC) anti-CD15 and anti-CD33 (for myeloid cells), Texas Red conjugated anti-CD3 (for T-lymphocytes) and allophycocyanin-cyanin7-conjugated (APC-Cy7) anti-CD19 (for B-lymphocytes) were used for sorting (BD Biosciences, Milan, Italy). Staining of healthy donor BM cells was performed in standard conditions, and sorting was performed using FACSAria (BD Biosciences). Data were analyzed using FACS Diva Software (v8.0.1, BD Biosciences). 

### 4.3. Animals

All mouse experiments were performed as previously described [24] and under the regulations, licenses and local ethical review from Queen Mary University of London (PPL license 70/8269) following UK Home Office Animals (Scientific Procedures) Act 1986. Briefly, C57bl6 females were bred with males of the same strain at the end of the daily light cycle and checked for vaginal plugs the following morning. The day of the vaginal plug was considered as embryonic age 0.5 dpc (days post coitum). Pregnant females were then sacrificed by cervical dislocation at desired gestational age for collecting embryos.

### 4.4. In Situ Hybridization 

Expression analysis by in situ hybridization was performed as previously described [24]. Briefly, paraffin sections of different stages embryos and fetuses were deparaffinized and hybridized overnight with *Nipbl* and *Smc1a* mouse DIG-labelled (Roche, Basel, Switzerland) anti-sense probes as reported. 

### 4.5. Reverse-Transcription PCR and Real-Time Quantitative-PCR Assays 

RNA was either available in the biobank or extracted from bone marrow and peripheral blood cells of healthy individuals with TRIZOL (Sigma, Mendota Heights, MN, USA) following manufacturer’s protocol. Superscript II enzyme (Thermo Fisher Scientific, Waltham, MA, USA) was used for cDNA synthesis. Quantitative RT-PCR experiments were performed as previously described [26] using Light Cycler 480 II with Universal Probe Master system (Roche, Basel, Switzerland). Primers and probes were selected according to the Software Probe Finder (Roche, Basel, Switzerland) and are reported in Table S9. Assays (primers and UPL probe matching) were designed using the dedicated UPL probe finder software v2.52 (release on 5 March 2017), available at Universal ProbeLibrary Assay Design Center (https://qpcr.probefinder.com). The coding alternative isoform transcripts contemporarily were selected, when present. Assays were then confirmed with the new software release v2.53 (release on 25 June 2017, database update: h_sap-Ensembl v89_38). In Table S9, we report the correspondent gene accession numbers, included in design and annotated in Nucleotide database (NCBI). The recognized amplicons were analyzed with the two genome browsers BLAST (https://blast.ncbi.nlm.nih.gov) and UCSC (https://genome.ucsc.edu/) respectively, confirming that these assays are specific (not recognizing unspecific or redundant targets) and detect all the protein coding transcript. *ABL1* gene was used as reference [40,41]. Four independent replicates were performed for each assay. Data have been shown as fold change, calculated as 2^−ΔΔ*C*t^ using the progenitor fraction (c-KIT+) as the calibrator sample (2^−ΔΔ*C*t^ = 1) [40]. For quantitative RT-PCR, data were statistically analyzed applying a two-tailed *t*-test setting *p* ≤ 0.05 as significant and indicated with symbol *.

## 5. Conclusions

In conclusion, the overall findings here presented point to tissues which should be particularly sensitive to the defects in cohesin genes, starting from crucial developmental windows, to postnatal life when the mantainance of those tissues homeostasis is required. This expression data may represent a first core reference to decode and interpret clinical findings displayed by patients with different CdLS subtypes.

## Supplementary Materials

Supplementary materials can be found at http://www.mdpi.com/1422-0067/19/2/438/s1.
