# Supplementary material for: Rings and Bricks: Expression of Cohesin Components is Dynamic during Development and Adult Life
Source: Int J Mol Sci. 2018 Feb 1;19(2):438. doi: 10.3390/ijms19020438 (PMC5855660; doi:10.3390/ijms19020438)

Table S1. Gene expression (2-ΔΔCt)

	NIPBL	*SMC1A*	*HDAC8*	*SMC3*	*RAD21*
**Heart**	1,00	1,00	1,00	1,00	1,00
**Adrenal**	3,30	1,45	2,98	1,25	0,65
**Salivary**	0,89	1,05	0,97	1,34	0,27
**Thyroid**	6,17	2,02	1,08	2,54	0,83
**Kidney**	0,14	1,01	1,23	0,89	0,38
**Small intestine**	5,50	2,81	1,18	2,03	0,53
**Stomach**	3,26	1,88	1,43	1,78	0,63
**Trachea**	1,49	1,26	0,97	1,34	0,30
**Lung**	0,26	1,33	1,19	1,30	0,28
**Fetal brain**	0,10	1,09	0,91	1,09	0,17
**Brain**	0,89	1,79	3,25	1,34	3,54
**Cerebellum**	89,16	8,23	6,56	9,45	3,06
**Fetal liver**	1,26	9,36	3,25	9,34	1,55
**Thymus**	41,28	7,83	2,08	6,89	2,36
**Spleen**	211,25	32,02	21,39	31,20	58,08
**Bone marrow**	61,18	9,50	1,68	8,05	3,35
**Peripheral blood**	57,76	7,03	0,89	7,56	2,48

Table S2. Comparison of *NIPBL* expression among original data and public datasets

	Data (2-ΔΔCt)	GTEx (TPM)	FANTOM 5 Project (TPM)	The Human Protein Atlas (TPM)
**Heart**	1,00	4,833	0,6	2,0
**Adrenal**	3,30	10,295	-	3,0
**Salivary**	0,89	15,070	-	2,0
**Thyroid**	6,17	22,655	-	3,0
**Kidney**	0,14	5,913	0,6	2,0
**Small intestine**	5,50	15,410		2,0
**Stomach**	3,26	10,995	-	-
**Trachea**	1,49	-	-	-
**Lung**	0,26	20,640	2,0	1,0
**Fetal brain**	0,10	-	-	-
**Brain**	0,89	-	1,0	-
**Cerebellum**	89,16	18,280	3,0	2,0
**Fetal liver**	1,26	-	-	-
**Thymus**	41,28	-	-	-
**Spleen**	211,25	17,455	2,0	-
**Bone marrow**	61,18	-	1,0	2,0
**Peripheral blood**	57,76	8,536	-	-

Table S3. Comparison of *SMC1A* expression among original data and public datasets

	Data (2-ΔΔCt)	GTEx (TPM)	FANTOM 5 Project (TPM)	The Human Protein Atlas (TPM)
**Heart**	1,00	8,0	19,0	2,0
**Adrenal**	1,45	13,0	-	3,0
**Salivary**	1,05	19,0	-	3,0
**Thyroid**	2,02	23,0	-	3,0
**Kidney**	1,01	9,0	21,0	3,0
**Small intestine**	2,81	-	-	3,0
**Stomach**	1,88	19,0	-	-
**Trachea**	1,26	-	-	-
**Lung**	1,33	24,0	12,0	2,0
**Fetal brain**	1,09	-	-	-
**Brain**	1,79	-	7,0	-
**Cerebellum**	8,23	30,0	47,0	2,0
**Fetal liver**	9,36	-	-	-
**Thymus**	7,83	-	-	-
**Spleen**	32,02	27,0	23,0	1,0
**Bone marrow**	9,50	-	31,0	3,0
**Peripheral blood**	7,03	7,0	-	-

Table S4. Comparison of *HDAC8* expression among original data and public datasets

	Data (2-ΔΔCt)	GTEx (TPM)	FANTOM 5 Project (TPM)	The Human Protein Atlas (TPM)
**Heart**	1,00	9,0	6,0	2,0
**Adrenal**	2,98	24,0	-	3,0
**Salivary**	0,97	31,0	-	2,0
**Thyroid**	1,08	39,0	-	2,0
**Kidney**	1,23	13,0	10,0	3,0
**Small intestine**	1,18	37,0	-	3,0
**Stomach**	1,43	23,0	-	-
**Trachea**	0,97	-	-	-
**Lung**	1,19	36,0	7,0	2,0
**Fetal brain**	0,91	-	-	-
**Brain**	3,25	-	4,0	-
**Cerebellum**	6,56	42,0	30,0	1,0
**Fetal liver**	3,25	-	-	-
**Thymus**	2,08	-	-	-
**Spleen**	21,39	39,0	11,0	-
**Bone marrow**	1,68	-	8,0	1,0
**Peripheral blood**	0,89	8,0	-	-

Table S5. Comparison of *SMC3* expression among original data and public datasets

	Data (2-ΔΔCt)	GTEx (TPM)	FANTOM 5 Project (TPM)	The Human Protein Atlas (TPM)
**Heart**	1,00	2,492	7,0	2,0
**Adrenal**	2,98	9,960	-	3,0
**Salivary**	0,97	6,215	-	3,0
**Thyroid**	1,08	8,913	-	3,0
**Kidney**	1,23	4,551	14,0	2,0
**Small intestine**	1,18	4,433	-	2,0
**Stomach**	1,43	4,553	-	-
**Trachea**	0,97	-	-	-
**Lung**	1,19	6,130	11,0	2,0
**Fetal brain**	0,91	-	-	-
**Brain**	3,25	-	9,0	-
**Cerebellum**	6,56	8,354	24,0	1,0
**Fetal liver**	3,25	-	-	-
**Thymus**	2,08	-	-	-
**Spleen**	21,39	5,198	9,0	1,0
**Bone marrow**	1,68	-	8,0	3,0
**Peripheral blood**	0,89	1,270	-	-

Table S6. Comparison of *RAD21* expression among original data and public datasets

	Data (2-ΔΔCt)	GTEx (TPM)	FANTOM 5 Project (TPM)	The Human Protein Atlas (TPM)
**Heart**	1,00	25,0	-	2,0
**Adrenal**	2,98	70,0	-	3,0
**Salivary**	0,97	50,0	-	3,0
**Thyroid**	1,08	89,0	-	3,0
**Kidney**	1,23	26,0	-	3,0
**Small intestine**	1,18	51,0	-	3,0
**Stomach**	1,43	46,0	-	-
**Trachea**	0,97	-	-	-
**Lung**	1,19	78,0	-	3,0
**Fetal brain**	0,91	-	-	-
**Brain**	3,25	-	-	-
**Cerebellum**	6,56	76,0	-	3,0
**Fetal liver**	3,25	-	-	-
**Thymus**	2,08	-	-	-
**Spleen**	21,39	58,0	-	3,0
**Bone marrow**	1,68	-	-	3,0
**Peripheral blood**	0,89	28,0	-	-

Table S7. Organs affected in CdLS (frequently in red, intermediate frequency in orange and rarely in yellow) compared to expression levels of causative genes (abundant in red, intermediate in orange and scarce in yellow)

Organ affected	*NIPBL*	*SMC1A*	*HDAC8*	*SMC3*	*RAD21*
**Brain**	0,89	1,79	3,25	1,34	3,54
**Cerebellum**	89,16	8,23	6,56	9,45	3,06
**Stomach**	3,26	1,88	1,43	1,78	0,63
**Intestine**	5,50	2,81	1,18	2,03	0,53
**Heart**	1,00	1,00	1,00	1,00	1,00
**Kidney**	0,14	1,01	1,23	0,89	0,38
**Liver**	1,26	9,36	3,25	9,34	1,55
**Lung**	0,26	1,33	1,19	1,30	0,28

Table S8. Comparison of gene expression in mouse embryos (present data is highlighted in yellow: E13.5 and E15.5) with GXD database (E14.5)

	Nipbl			*Smc1a*		
	E13.5	***E14.5***	***E15.5***	***E13.5***	***E14.5***	***E15.5***
**Brain**	**NO**	YES	**YES**	**YES**	YES	**YES**
**Cerebellum**	**YES**	YES	**YES**	**YES**	YES	**YES**
**Ear**	**YES**	NO	**YES**	**YES**	YES	**YES**
**Face**	**YES**	NO	**YES**	**NO**	NO	**NO**
**Nose**	**YES**	YES	**YES**	**NO**	YES	**YES**
**Tooth**	**NO**	NO	**YES**	**-**	YES	**YES**
**Heart**	**YES**	NO	**YES**	**YES**	YES	**YES**
**Liver**	**NO**	NO	**YES**	**YES**	YES	**YES**
**Lung**	**YES**	NO	**YES**	**NO**	YES	**YES**
**Reprodutive system**	**-**	YES	**YES**	**-**	YES	**YES**

Table S9. Primers for real-time quantitative-PCR probes

Gene	*Forward Primer*	*Reverse Primer*	*Probe*	*Amplicon*	*Nucleotide Accession Number*	*Ensembl*
NIPBL	ctatgcgaacagcccaaaa	ttcaccttgcttactaccacattt	55	ctatgcgaacagcccaaaacttcctctccatcttccttaaaa aatgtggtagtaagcaaggtgaa	NM_015384.4+NM_133433.3	ENST00000282516.12, ENST00000448238.2
SMC1A	cgacatctagccctgaatctg	attaatgcgaggcccaaagt	78	cgacatctagccctgaatctgcaggaaaaatccaagctgg agagtgagctagccaactttgggcctcgcattaat	NM_006306.2 + NM_001281463.1	ENST00000375340.10, ENST00000322213.8
SMC3	tgcactgaatgatgagattcgt	ttaattctttcatttagcaactgtctg	18	tggagaaaaatccagacaattaagagatgctcagcaggatg caagagataaaatggaggatatcgaacgccaagt	NM_005445	ENST00000361804.4
RAD21	tcattaagataaagatggcttttcg	tcaaagtcatgaaattcttcaggt	38	tcattaagataaagatggcttttcggccaggtgtggttgac ctgcctgaggaaaatcgggaagcagcttataatgccatta ctttacctgaagaatttcatgactttga	NM_006265.2	ENST00000297338.6
HDAC8	gcactgcataagcagatgagg	tggagatgctgcagataagc	25	gcactgcataagcagatgaggatagttaagcctaaagtgg cctccatggaggagatggccaccttccacactgatgcttat ctgcagcatctcca	NM_001166418	ENST00000439122.6,ENST00000373573.7,E NST00000373589.8,ENST00000373554.5,EN ST00000373556.7,ENST00000373559.8,ENST 00000373571.5, ENST00000373583.5,

Supplementary Figure S1

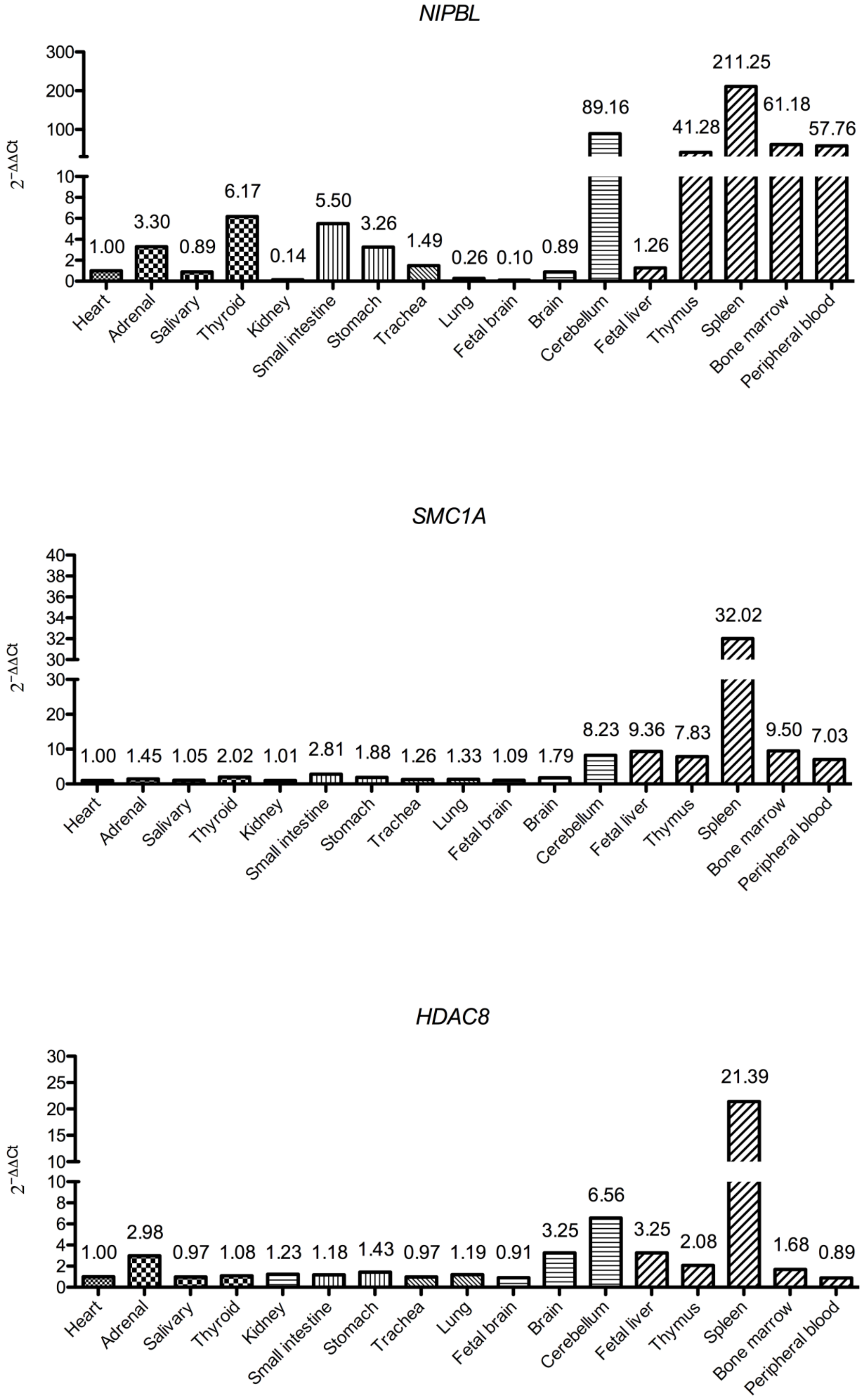

Supplementary Figure S2

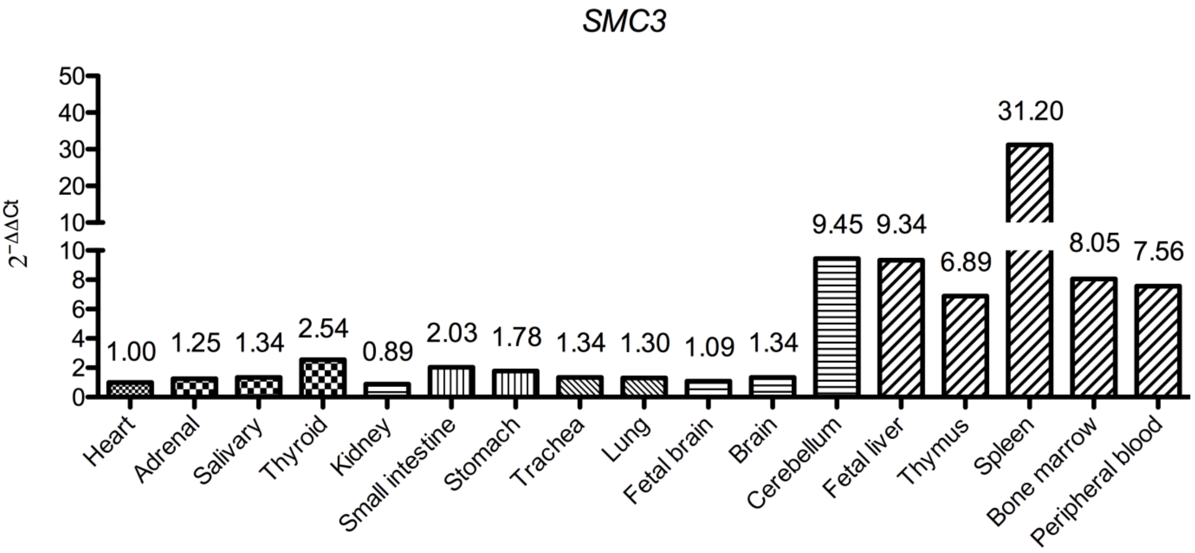

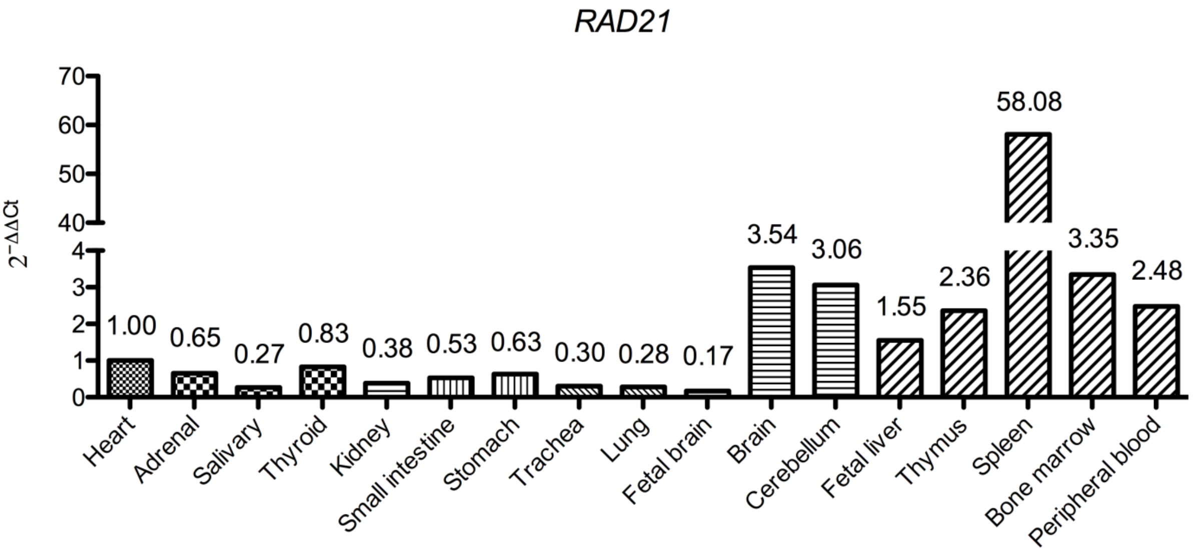

Supplement: Supplementary file 1 [file ijms-19-00438-s001.pdf]
